# Forefront studies on HTLV-1 oncogenesis

**DOI:** 10.3389/fmicb.2013.00156

**Published:** 2013-06-18

**Authors:** Renaud Mahieux, Toshiki Watanabe

**Affiliations:** ^1^Equipe Oncogenèse Rétrovirale, CIRI Inserm U1111-CNRS UMR5308Paris, France; ^2^Equipe Labellisée “Ligue Nationale Contre le Cancer”, CIRI Inserm U1111-CNRS UMR5308Paris, France; ^3^International Center for Research in Infectiology, INSERM U1111 - CNRS UMR5308Paris, France; ^4^Ecole Normale Supérieure de LyonLyon, France; ^5^Université Lyon 1Lyon, France; ^6^Laboratory of Tumor Cell Biology, Department of Medical Genome Sciences, Graduate School of Frontier Sciences, The University of TokyoTokyo, Japan

Almost 40 years ago, Takatsuki et al. recognized the existence of a peculiar T cell leukemia in Kyoto, Japan that they named Adult T Leukemia (ATL). They reported a series of 13 patients in 1976 (Uchiyama et al., 1977). In 1980, the group of Gallo reported the discovery of a human oncogenic retrovirus that they named Human T cell Leukemia Virus type 1 (HTLV-1) in cells obtained from two US patients classified as mycosis fungoides and Sezary syndrome (Poiesz et al., 1980), but who were, in retrospect, probably suffering from ATL [for an historical perspective see (Takatsuki, 2005)]. Shortly after, the groups of Hinuma (Miyoshi et al., 1981) and of Yoshida (Yoshida et al., 1982) uncovered the presence of HTLV-1 in cells obtained from ATL patients. In 1985–1986, two groups independently reported that a neurological disease named HTLV-1 Associated Myelopathy/Tropical Spastic Paraparesis (HAM/TSP) was also caused by HTLV-1 (Gessain et al., 1985; Osame et al., 1986). Since then, other inflammatory diseases (uveitis, infective dermatitis) have also been linked to this viral infection. Other members of the HTLV family (i.e., HTLV-2, HTLV-3, and HTLV-4 have also now been reported, none of them being clearly associated so far with an oncogenic process or a neurodegenerative disease (Kalyanaraman et al., 1982; Calattini et al., 2005; Wolfe et al., 2005).

Almost 10 years ago, ours colleagues Kuan Teh Jeang and Mitsuaki Yoshida organized a special issue on HTLV infection in Oncogene. In setting up this issue, we cannot forget the memory our friend Teh.

We called upon the expertise of different research groups from Europe, Japan, and USA. However, we regret that the format of this issue prevented us from soliciting many other colleagues. The following reviews will deal with many fascinating aspects of viral cycle, but summarizes also new approaches that should allow a better integrated research.

A first group of articles provides information about HTLV-1 epidemiology and associated-pathogenesis. The article from Gessain and Cassar provides an updated view on HTLV-1 distribution, based on data obtained from 1.5 billion individuals originating from endemic areas (Gessain and Cassar, 2012). Iwanaga et al. focused their review on ATL epidemiology and show its peculiar characteristic [age at onset, risk factor, proviral load, etc. (Iwanaga et al., 2012)]. Yamano and Sato provide an interesting perspective on HAM/TSP physiopathology, and remind us that optimal therapeutic treatments are still lacking for those patients (Yamano and Sato, 2012). The review by Kamoi ad Mochizuki summarizes our current knowledge on HTLV-1 uveitis, which is the most common cause of uveitis in endemic areas (Kamoi and Mochizuki, 2012). Going deeper in the pathological mechanisms linked to HTLV-1 infection, Yamagishi and Watanabe summarize recent data showing that ATL cells express abnormally low levels of a cellular oncosuppressor miRNA and display some epigenetic changes on the promoter of genes critical for cell cycle (Yamagishi and Watanabe, 2012).

A second group of articles summarizes the interaction between the virus and the host's cells. Before causing diseases, HTLV-1 has to enter the cell. However, the mechanisms of HTLV-1 transmission and cell entry have remained elusive for a long period of time. Pique and Jones have summarized recent insights about those mechanisms both at the cell level but also between individuals (Pique and Jones, 2012). HTLV-1 associated diseases are linked to the fact that HTLV-1 evades both adaptative and innate immune responses. Kannagi et al. provide us with an exciting review, which explains us how the virus evades the interferon response, but also that dysfunction of the CTL response might be a risk factor for disease development in infected carriers (Kannagi et al., 2012).

A third group of articles reports data on individual viral proteins that play important roles in the viral cycle and/or in pathogenesis. Nakano and Watanabe remind us the important role played by Rex, which uses cellular pathways to export unspliced or singly spliced viral mRNAs in the cell cytoplasm, therefore allowing expressing of structural proteins (Nakano and Watanabe, 2012). Currer et al., Zhao and Matsuoka focused their attention on Tax and HBZ, two viral proteins that play important roles in the control of viral transcription and oncogenesis (Currer et al., 2012; Zhao and Matsuoka, 2012). Finally, Bai and Nicot provide an overview on 4 auxiliary viral proteins (p12, p8, p30, and p13), which are required for establishing a persistent infection *in vivo* (Bai and Nicot, 2012).

Finally, Duc Dodon and colleagues remind us that studying HTLV-1 pathogenesis requires animal models (Dodon et al., 2012). Rabbits, rats, transgenic mice, and monkeys have been used in the past. However, recent approaches using humanized mice might represent an interesting alternative for studying HTLV-1 associated diseases.
